# Sex-specific differences in sleep-disordered breathing and nocturnal hypoxemia in chronic thromboembolic pulmonary hypertension and chronic thromboembolic pulmonary disease

**DOI:** 10.3389/fcvm.2022.966973

**Published:** 2022-10-17

**Authors:** Hui-Ting Li, Ping Yuan, Qin-Hua Zhao, Su-Gang Gong, Rong Jiang, Jin-Ling Li, Hong-Ting Liu, Hong-Ling Qiu, Wen-Hui Wu, Ci-Jun Luo, Jing He, Lan Wang, Jin-Ming Liu

**Affiliations:** Department of Cardio-Pulmonary Circulation, School of Medicine, Shanghai Pulmonary Hospital, Tongji University, Shanghai, China

**Keywords:** chronic thromboembolic pulmonary disease, chronic thromboembolic pulmonary hypertension, nocturnal hypoxemia, sleep-disordered breathing, sex difference

## Abstract

**Objective:**

Although chronic thromboembolic pulmonary hypertension (CTEPH) and chronic thromboembolic pulmonary disease (CTEPD) are known to be accompanied by symptoms associated with sleep-disordered breathing (SDB) and nocturnal hypoxemia, the sex-specific differences of SDB and nocturnal hypoxemia in patients with CTEPH and CTEPD remain unknown.

**Methods:**

Between July 2020 and August 2022, data were retrieved from 57 males and 63 female patients with CTEPH and CTEPD who underwent sleep study at Shanghai Pulmonary Hospital. Nocturnal mean SpO_2_ (mean SpO_2_) < 90% was defined as nocturnal hypoxemia. Logistic and linear regression analysis was performed to assess the predictive value of sleep study indices to hemodynamic parameters. Receiver operating characteristic (ROC) curve was applied to analyze the specific parameters to predict the risk of CTEPH.

**Results:**

SDB was similarly present in males and females, and both sexes predominantly had obstructive sleep apnea (OSA); more women were diagnosed with nocturnal hypoxemia (32 vs. 7%, *p* = 0.002). SaO_2_ was negatively associated with mean pulmonary arterial pressure (mPAP) in men (*p* < 0.001), whereas the ratio of nocturnal SpO_2_ < 90% of the total monitoring time (T90%) was positively correlated with mPAP. Mean SpO_2_ was an independent predictor for pulmonary vascular resistance and cardiac output in women (*p* = 0.001, *p* < 0.001, *p* = 0.001, respectively). T90%, SaO_2_, and minimal SpO_2_ were combined to develop a new composite parameter: hypoxemia scoring index (HSI). ROC curve analysis indicated that HSI levels of 0.55 could discriminate CTEPH from CTEPD with a sensitivity of 92.3% and specificity of 87.5% in female patients (an area under the curve, 0.937; 95% CI: 0.879–0.995, *p* < 0.001).

**Conclusion:**

Sex-specific nocturnal hypoxemia was present in patients with CTEPH or CTEPD. In female patients, the HSI showed high capacity for predicting the risk of CTEPH.

**Clinical trials registration:**

Registry: chictr.org.cn; Identifier: ChiCTR-DDD-16009406.

## Introduction

Chronic thromboembolic pulmonary hypertension (CTEPH) is a progressive pulmonary vascular disease characterized by persistent obstruction of pulmonary arteries, progressive vascular remodeling, and elevated mean pulmonary arterial pressure (mPAP). Approximately 70% of patients with CTEPH had prior venous thromboembolism (1). According to a recent population-based study conducted in England, women may be more prone to developing CTEPH than men (2). The sex-specific incidence rate, mortality, and some other clinical data regarding the pathogenesis of CTEPH were also supported by previous studies (3–11). In that, women with CTEPH underwent pulmonary endarterectomy less frequently than men, especially at low-volume centers. Furthermore, they had a lower prevalence of cardiovascular risk factors and were less often exposed to additional cardiac surgery procedures, which may explain the better long-term survival of women (12). Another study also showed that sex-specific differences existed not only in cardiopulmonary function but also in event-free survival rate between the two sexes in inoperable patients with CTEPH (13).

Chronic thromboembolic pulmonary disease (CTEPD) is characterized by the presence of residual pulmonary vascular obstruction by chronic thromboembolic material in the pulmonary arteries without pulmonary hypertension (PH) at rest (14). Patients with CTEPH and CTEPD frequently have other accompanying symptoms such as, snoring, mouth breathing, and insomnia which are associated with sleep-disordered breathing (SDB) and nocturnal hypoxemia (15). SDB, mainly obstructive sleep apnea (OSA), is generally prevalent in the population with CTEPH (15, 16). Some studies also have indicated that nocturnal hypoxemia was also highly prevalent in patients with CTEPH (17, 18). Sex differences in upper airway structure, fat distribution and arousal response to apneas may contribute to a greater susceptibility to OSA in male patients (19).

To our knowledge, no comprehensive analysis of sex differences of SDB and nocturnal hypoxemia in CTEPH and CTEPD has been carried out thus far. Our study aimed to clarify the sex differences of SDB and nocturnal hypoxemia in CTEPH and CTEPD and investigate whether “sex-specific” SDB or nocturnal hypoxemia is related with the hemodynamics or occurrence risk of CTEPH.

## Methods

### Study population and design

This was a single-center, retrospective study. One hundred and fifty-four patients with CTEPH and CTEPD who systematically underwent echocardiography, right heart catheterization (RHC), and sleep study at Shanghai Pulmonary Hospital from July 2020 to August 2022 were consecutively recruited. The diagnosis of CTEPH and CTEPD was based on the latest European Society of Cardiology (ESC) criteria (20). All patients were newly diagnosed and underwent RHC within 1 week prior to diagnosis, and they were clinically stable (New York Heart Association [NYHA] class I-IV) and aged >18 years. The exclusion criteria were as follows: (i) aged >80 years; (ii) daytime resting SpO_2_ < 90%; (iii) domestic use of nocturnal positive airway pressure therapy; (iv) comorbidities including chronic obstructive pulmonary disease or severe left heart disease; (v) sleep duration < 3 h or having some sleep disorder; (vi) patients who underwent balloon pulmonary angioplasty or pulmonary endarterectomy;and (vii) patients with upper airway stenosis and a family history of sleep apnea. This study was conducted in accordance with the tenets of the amended Declaration of Helsinki. The local Institutional Ethics Committee of Shanghai Pulmonary Hospital approved the study protocol (L22-198). All patients provided written informed consent.

### Clinical assessments

Demographic information, pulmonary function, and echocardiography variables were obtained. RHC was performed in all hospitalized patients. The baseline hemodynamic parameters of mean pulmonary arterial pressure (mPAP), pulmonary vascular resistance (PVR), right atrial pressure (RAP), cardiac output (CO), and cardiac index (CI) were measured by RHC (21).

### Sleep studies

Sleep studies were performed using the Ventilatory Effort Recorder (Alice NightOne, Philips, NY, USA) in the hospital and evaluated according to standard recommendations (22). Signals obtained included nasal pressure flow, abdominal and thoracic movements, pulse and oxygen saturation, snoring, and physiological data. SDB severity was graded according to the apnea-hypopnea index (AHI), and sleep apnea was defined as an AHI of ≥5/h. OSA was defined as the cessation of airflow in the presence of thoracic and abdominal wall motion, whereas CSA was defined as cessation of both airflow and thoracic and abdominal wall motion. In accordance with the standard guidelines (22), SDB was defined as mild (AHI ≥5 to < 15/h), moderate (AHI ≥15 to < 30/h), or severe (AHI ≥30/h). Nocturnal mean SpO_2_ (mean SpO_2_) < 90% was defined as nocturnal hypoxemia. The severity of nocturnal hypoxemia was determined by the ratio of nocturnal SpO_2_ < 90% of the total monitoring time (T90%), and desaturation was defined as T90% exceeding 10%. The nocturnal minimal SpO_2_ (min SpO_2_) was also recorded.

### Statistical analysis

SPSS 23.0 software (IBM Corporation, Armonk, NY, USA) was used for all data analysis. Data are expressed as mean ± standard deviation or median (interquartile range). Differences between groups were assessed using the Student's *t*-test or one-way ANOVA for normally distributed continuous variables and the Mann–Whitney-U test for non-normally distributed variables. Chi-squared test or Fisher exact test was used to compare categorical variables.

Binary logistic regression was used to explore possible predictors for nocturnal hypoxemia, desaturation, and CTEPH. A stepwise selection procedure was used to identify independent predictors with p-to-enter of ≤ 0.10 and p-to-remove of ≥0.15. Multivariate relationships between hemodynamic parameters and nocturnal hypoxemia were examined by linear regression (enter method), with *p* < 0.05 to enter and *p* > 0.1 to remove. Covariate diagnostic method was used to screen the entry variables.

The receiver operating characteristic (ROC) curve was used to assess the ability of hypoxemia indicators and HSI to distinguish CTEPH and CTEPD. The sensitivity and specificity were calculated. Statistical tests were two-tailed, and *p* < 0.05 was considered to indicate statistically significant differences.

## Results

### Characteristics of participants

**Figure 1:**
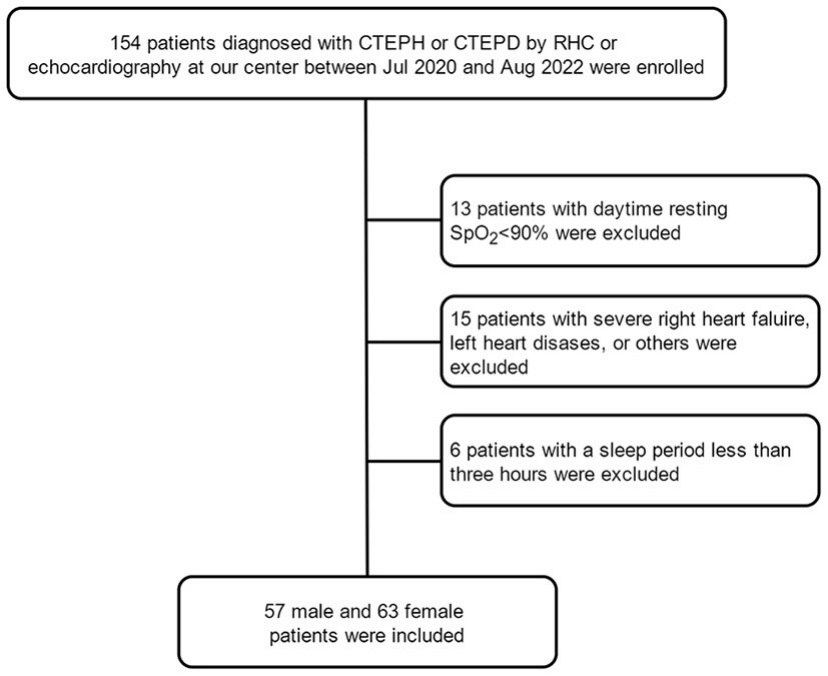
Flow chart of enrolled patients. CTEPD, chronic pulmonary thromboembolic disease; CTEPH, chronic thromboembolic pulmonary hypertension.

In our study, 72 and 48 patients with CTEPH and CTEPD, respectively, met the inclusion criteria and were enrolled. There were 57 male and 63 female patients (Figure 1). The baseline clinical characteristics of male and female patients were compared, as shown in Table 1. It was showed in the Supplementary material. Female patients had the same age and BMI as male patients (*p* = 0.058, *p* = 0.207, respectively). Other clinical variables such as NYHA class, 6-min walk distance (6MWD), N-terminal pro-brain natriuretic peptide (NT-proBNP) were not different between the two groups (all *p* > 0.05). Female patients had lower SaO_2_ and PaO_2_ than male patients (*p* = 0.002, *p* = 0.001, respectively). With regard to pulmonary function, female patients showed higher percentage of predicted (%predicted) residual volume (RV, %predicted) and percentage of RV and total lung capacity (TLC) (RV/TLC, %) (*p* = 0.004, *p* < 0.001, respectively) and lower forced expiratory volume in 1 s (FEV_1_), forced vital capacity (FVC), TLC, single-breath carbon monoxide diffusing capacity (DLco-SB), and percentage of predicted (%pred) DLco-SB (DLco-SB, %predicted) than male patients (*p* < 0.001, *p* < 0.001, *p* < 0.001, *p* < 0.001, *p* = 0.006, respectively). For echocardiographic and hemodynamic variables, there was no difference between the two groups (all *p* > 0.05).

**Table 1:** Demographic and baseline clinical characteristics of all participants.

**Variable**	**Male (*n* = 57)**	**Female (*n* = 63)**	***p*-value**
Age, years	59.09 ± 14.40	63.40 ± 9.44	0.058
BMI, kg·m^−2^	24.00 ± 3.53	24.83 ± 3.60	0.207
**NYHA class**, ***n*** **(%)**			
I	32 (57)	38 (60)	0.643
II	12 (21)	13 (21)	0.955
III	11 (19)	10 (16)	0.622
IV	2 (3)	2 (3)	1.000
Smoking/second smoking, *n* (%)	48 (84)	45 (71)	0.094
**Comorbidities**, ***n***			
Diabetes	5	11	0.315
Systemic hypertension	9	15	0.852
6 MWD, m	440 (350–504)	420 (322–455)	0.271
NT-proBNP, pg/ml	142.6 (33.4–663.0)	135.9 (65.0–629.2)	0.497
**Arterial blood ga**s			
SaO_2_, %	95.5 (94.5–97.1)	94 (91.5–95.9)	0.002
PaO_2_, mmHg	75.0 (69.0–87.7)	68 (62.0–75.0)	0.001
**Echocardiographic data**			
PASP, mmHg	57.04 ± 27.31	53.85 ± 27.76	0.562
TAPSE, cm	1.98 ± 0.40	1.96 ± 0.37	0.732
TAPSE/SPAP, %	0.04 (0.02–0.05)	0.04 (0.03–0.06)	0.267
RA area, cm^2^	15.50 (12.95–20.45)	15.70 (12.50–18.85)	0.621
LVEF < 50%, *n* (%)	0 (0)	0 (0)	-
**Lung function data**			
FEV_1_, L	2.65 ± 0.87	1.80 ± 0.39	< 0.001
FEV_1_, %predicted	91.45 (76.70–102.1)	81.20 (72.70–90.45)	0.019
FVC, L	3.41 ± 0.94	2.31 ± 0.47	< 0.001
FEV_1_/FVC, %	77.71 ± 9.64	77.95 ± 7.44	0.888
RV, L	2.41 (2.10–2.84)	2.39 (1.99–2.84)	0.582
RV, %predicted	112.7 (104.8–142.2)	136.5 (123.8–152.5)	0.004
TLC, L	5.97 (4.93–6.50)	4.64 (4.14–5.15)	< 0.001
TLC, %predicted	100.1 (93.5–107.9)	105.3 (97.8–113.8)	0.077
RV/TLC, %	42.44 (37.01–48.69)	52.70 (47.54–55.62)	< 0.001
DL_CO_-SB, ml/min/mmHg	19.29 (15.13–22.38)	15.10 (13.03–16.18)	< 0.001
DL_CO_-SB, %predicted	87.05 (77.98–105.90)	82.40 (71.43–90.00)	0.006
DL_CO_/VA, ml/min/mmHg	3.55 ± 0.90	3.64 ± 0.74	0.215
**RHC data**			
SvO_2_, mmHg	63.60 ± 9.74	62.22 ± 8.26	0.478
mPAP, mmHg	33.77 ± 14.13	39.16 ± 14.23	0.065
RAP, mmHg	4.44 ± 1.91	4.99 ± 3.85	0.653
PVR, wood unit	5.94 ± 4.07	7.70 ± 4.13	0.053
CO, L/min	5.14 ± 1.26	4.99 ± 1.43	0.612
CI, L/min/m^2^	2.85 (2.33–3.45)	3.17 (2.54–3.49)	0.349

Values are depicted as mean ± standard deviation or median (interquartile range) for continuous variables depending on normality distribution of the data. Categorical data are expressed as number of patients (%). AHI, apnea-hypopnea index; BMI, Body Mass Index; CI, cardiac Index; CO, cardiac output; CTEPD, chronic pulmonary thromboembolic disease; CTEPH, chronic thromboembolic pulmonary hypertension; DLCO-SB, single-breath carbon monoxide diffusing capacity; DLCO/VA, diffusing capacity divided by the alveolar volume; FEV1, forced expiratory volume in 1 s; FVC, forced vital capacity; LVEF, left ventricular ejection fraction; mPAP, mean pulmonary arterial pressure; NT-proBNP, N-terminal pro-brain natriuretic peptide; NYHA, New York Heart Association functional class; PVR, pulmonary vascular resistance; RA, right room; RAP, right atrial pressure; RV, residual volume; PASP, systolic pulmonary artery pressure; SvO_2_, mixed venous oxygen saturation; TAPSE, tricuspid value systolic displacement; TLC, total lung capacity; 6MWD, 6-minute walking distance.

### Sleep studies

#### Sex difference of SDB in patients

**Figure 2:**
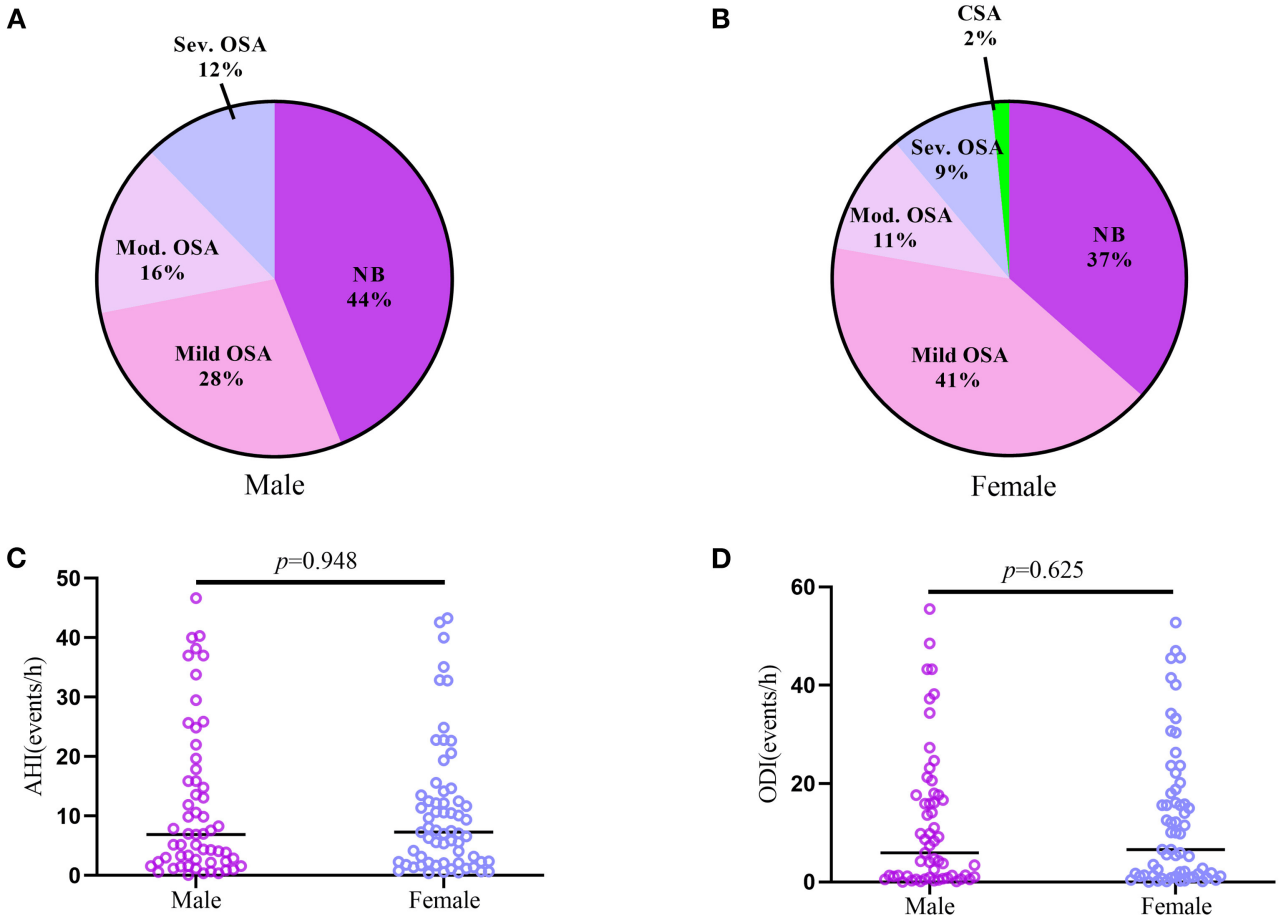
The difference of SDB between Male and Female patients with CTEPH and CTEPD. **(A,B)** The prevalence and type of SDB in the two groups. **(C,D)** Male (*n* = 57) and Female (*n* = 63) patients had no different AHI or ODI. AHI, apnea-hypopnea index; CSA, central sleep apnea; CTEPD, chronic pulmonary thromboembolic disease; CTEPH, chronic thromboembolic pulmonary hypertension; ODI, Oxygen desaturation index; OSA, obstructive sleep apnea.

Except for one female patient who showed pure CSA, SDB in the two groups was characterized by OSA (Figure 2). The prevalence of OSA among male and female patients was the same (*p* = 0.412); 16 male and 26 female patients were documented with mild OSA (*p* = 0.483); and 16 male and 13 female patients were documented with moderate-to-severe OSA (*p* = 0.342). There was no difference in AHI between the male and female groups [6.90 (2.25–16.90) vs. 7.30 (2.20–13.50), *p* = 0.948]; furthermore, male patients also had similar oxygen desaturation index (ODI) as female patients [5.90 (0.95–17.70) vs. 6.60 (1.20–18.80), *p* = 0.625].

#### Sex difference of nocturnal hypoxemia in patients

**Figure 3:**
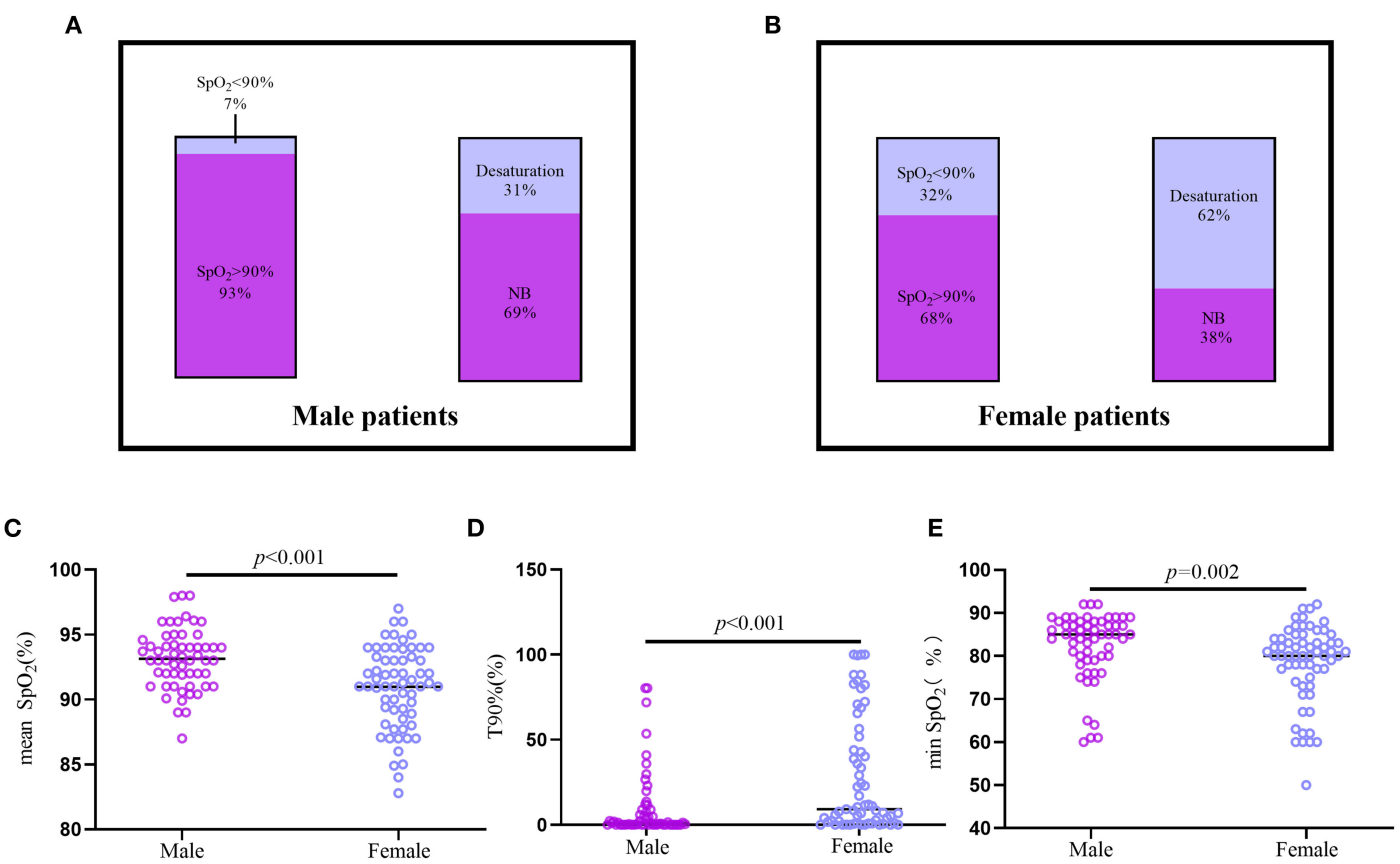
The difference of nocturnal hypoxemia between Male and Female patients with CTEPH and CTEPD. **(A,B)** The prevalence of nocturnal hypoxemia and desaturation in male (*n* = 57) and female (*n* = 63) patients. **(C–E)** Male CTEPH patients had higher mean SpO_2_, min SpO_2_ and lower T90% than the female CTEPH patients. CTEPD, chronic pulmonary thromboembolic disease; CTEPH, chronic thromboembolic pulmonary hypertension; mean SpO_2_, nocturnal mean SpO_2_; min SpO_2_, minimal SpO_2_; T90%, the ratio of nocturnal SpO_2_ below 90% of the total monitoring time.

Female patients showed more severe nocturnal hypoxemia than male patients (Figure 3). Twenty females were diagnosed with nocturnal hypoxemia, whereas only four male patients had nocturnal hypoxemia (*p* = 0.002). Thirty-one female and 13 male patients were found to have desaturation (*p* = 0.003). The T90% in female patients was higher than that in male patients [9.18 (1.12–51.85) vs. 1.03 (0.02–8.91), *p* < 0.001], whereas the mean SpO_2_ and min SpO_2_ were both lower in females [90.97 ± 3.13 vs. 93.13 ± 2.40, *p* < 0.001; 80 (75–84) vs. 85 (80–88), *p* = 0.002, respectively].

#### Predictors of nocturnal hypoxemia in male and female patients with CTEPH and CTEPD

Age, PaO_2_, SaO_2_, and mPAP were revealed to be correlated with desaturation in male patients through univariate regression analysis (all *p* < 0.05). Multivariate regression analysis revealed that PaO_2_ was an independent predictor for desaturation in the male group (*p* = 0.007) (Table 2).

**Table 2:** Predictors of nocturnal hypoxemia and desaturation in male and female patients with CTEPH and CTEPD.

**Group**	**Categorical variables**	**Independent Variables**	**Univariate analysis**		**Multivariate analysis**	
			**OR (95% CI)**	***p-*value**	**OR (95% CI)**	***p-*value**
Male	Desaturation	Age	1.096 (1.009, 1.191)	0.030	-	-
		PaO_2_	0.874 (0.803, 0.951)	0.002	-	-
		SaO2	0.649 (0.484, 0.871)	0.004	0.655 (0.480, 0.893)	0.007
		mPAP	1.080 (1.018, 1.146)	0.011	-	-
Female	Nocturnal hypoxemia	PaO_2_	0.946 (0.899, 0.996)	0.035	-	-
		SaO2	0.872 (0.761, 0.999)	0.049	-	-
		PASP	1.024 (1.003, 1.046)	0.026	-	-
		CO	0.524 (0.304, 0.906)	0.021	-	-
		PVR	1.220 (1.030, 1.444)	0.021	-	-
		DLco-SB	0.738 (0.570, 0.955)	0.021	0.727 (0.537, 0.984)	0.039
	Desaturation	PaO_2_	0.949 (0.907, 0.993)	0.022	-	-
		PASP	1.042 (1.014, 1.070)	0.003	-	-
		mPAP	1.075 (1.023, 1.129)	0.004	-	-
		CO	0.404 (0.206, 0.791)	0.008	-	-
		PVR	1.461 (1.154, 1.880)	0.002	1.509 (1.151, 1.979)	0.003

CI, confidence interval; CO, cardiac output; CTEPD, chronic pulmonary thromboembolic disease; CTEPH, chronic thromboembolic pulmonary hypertension; DLco-SB, single-breath carbon monoxide diffusing capacity; mPAP, mean pulmonary arterial pressure; OR, odd ratio; PASP, systolic pulmonary artery pressure; PVR, pulmonary vascular resistance.

Univariate logistic regression analysis demonstrated a relationship among PaO_2_, SaO_2_, PASP, CO, PVR, DLco-SB, and nocturnal hypoxemia in female patients (all *p* < 0.05). Multivariate logistic regression analysis indicated that DLco-SB was independently related with nocturnal hypoxemia (*p* = 0.039) (Table 2). PaO_2_, PASP, mPAP, CO, and PVR showed correlation with desaturation in female patients through univariate regression analysis (all *p* < 0.05). After multivariate regression analysis, PVR was shown to be independently correlated with desaturation in female patients (*p* = 0.003) (Table 2).

#### Hypoxemia-related indicators associated with PVR, mPAP, and CO in male and female patients with CTEPH and CTEPD

For male patients, univariate linear regression analysis showed that SaO_2_, mean SpO_2_ and T90% were correlated with mPAP (all *p* < 0.05), while multivariate linear regression analysis showed that only SaO_2_ was negatively associated with mPAP (*p* < 0.001) (Table 3).

**Table 3:** Hypoxemia-related indicators associated with PVR, mPAP and CO in male and female patients with CTEPH and CTEPD.

**Group**	**Dependent variable**	**Independent variable**	**Univariate analysis**		**Multivariate analysis**	
			**Beta**	***p-*value**	**Beta**	***p-*value**
Male	mPAP	SaO_2_	−0.524	< 0.001	−0.524	< 0.001
		Mean SpO_2_	−0.386	0.007	-	-
		T90%	0.451	0.001	-	-
Female	mPAP	PaO_2_	−0.293	0.041	-	-
		SaO2	−0.289	0.044	-	-
		Mean SpO_2_	−0.384	0.006	-	-
		T90%	0.461	0.001	0.461	0.001
	PVR	Mean SpO_2_	−0.554	< 0.001	−0.554	< 0.001
		T90%	0.496	0.001	-	-
	CO	Mean SpO_2_	0.476	0.001	0.476	0.001
		T90%	−0.419	0.005	-	-

CI, confidence interval; CO, cardiac output; CTEPD, chronic pulmonary thromboembolic disease; CTEPH, chronic thromboembolic pulmonary hypertension; mean SpO_2_, nocturnal mean SpO_2_; min SpO_2_, minimal SpO_2_; mPAP, mean pulmonary arterial pressure; OR, odd ratio; PVR, pulmonary vascular resistance; T90%, the ratio of nocturnal SpO_2_ below 90% of the total monitoring time.

In female patients, PaO_2_, SaO_2_, mean SpO_2_, and T90% were all correlated with mPAP through univariate linear regression analysis (all *p* < 0.05); after multivariate linear regression analysis, T90% was shown to be an independent predictor for mPAP (*p* = 0.001) (Table 3). Both mean SpO_2_ and T90% were correlated with PVR and CO through univariate linear regression analysis (all *p* < 0.05); multivariate linear regression analysis proved that mean SpO_2_ was independently correlated with PVR and CO (*p* < 0.001, *p* = 0.001, respectively) (Table 3).

#### A novel and sex-specific hypoxemia scoring index to distinguish CTEPH and CTEPD

**Figure 4:**
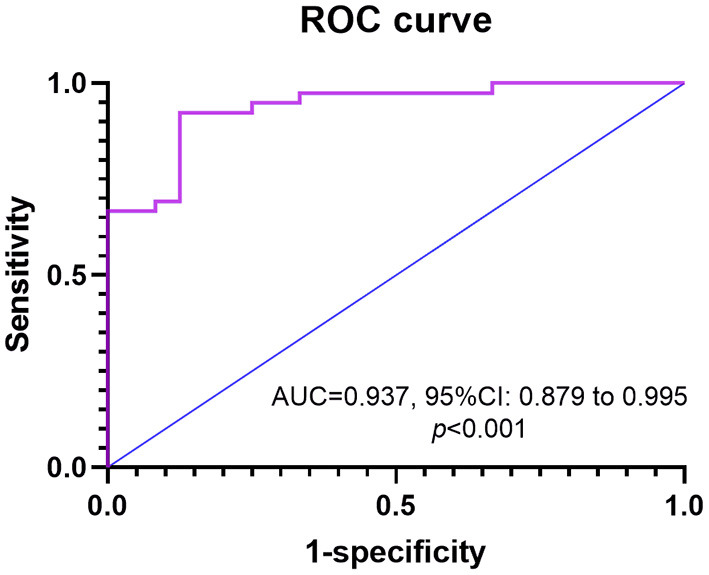
The ROC curve is shown for HIS as determined by hypoxemia factors in predicting CTEPH for female patients. AUC, an area under ROC curve; CTEPH, chronic thromboembolic pulmonary hypertension; HIS, hypoxemia scoring index; ROC, receiver operating characteristic.

We developed an HSI to identify CTEPH and CTEPD, for nocturnal hypoxia in male patients did not correlate with hemodynamics, so the HSI was only available for female patients. Univariate logistic regression analysis showed that mean SpO_2_, T90%, min SpO_2_, SaO_2_, and PaO_2_ were correlated with CTEPH (Table 4). Multivariate analysis was performed to construct the HSI. Stepwise logistic regression analysis revealed three variables that were independently significant: T90%, SaO_2_, and min SpO_2_. The equation for HSI was derived from the β-coefficients in the final model (Figure 4): HSI = 64.006 + 0.061 (T90%) - 0.609 (SaO_2_) - 0.092 (min SpO_2_).

**Table 4:** Hypoxemia-related indicators associated with female CTEPH.

**Variable**	**Univariate analysis**		**Multivariate analysis**		**β-coefficient**
	**OR (95% CI)**	***p-*value**	**OR (95% CI)**	***p-*value**	
Mean SpO_2_	0.686 (0.542, 0.869)	0.002	-	-	
T90%	1.070 (1.022, 1.119)	0.004	1.063 (1.015, 1.109)	0.010	0.061
Min SpO_2_	0.917 (0.849, 0.990)	0.026	0.912 (0.833, 0.997)	0.043	0.912
SaO_2_	0.598 (0.446, 0.801)	0.001	0.544 (0.365, 0.811)	0.003	−0.609
PaO_2_	0.893 (0.836, 0.954)	0.001	-	-	-

CI, confidence interval; CTEPH, chronic thromboembolic pulmonary hypertension; Mean SpO_2_, nocturnal mean SpO_2_; Min SpO_2_, minimal SpO_2_; OR, odd ratio; T90%, the ratio of nocturnal SpO_2_ below 90% of the total monitoring time.

Based on ROC curve analysis, HSI≥0.55 displayed a 92.3% sensitivity and 87.5% specificity with an area under ROC curve (AUC) of 0.937 [95% confidence interval (CI): 0.879–0.995, *p* < 0.001].

## Discussion

In our study, OSA was similarly and highly prevalent in male and female patients with CTEPH and CTEPD, whereas sex difference was only present in nocturnal hypoxemia, and female patients showed worse nocturnal hypoxemia than male patients. Nocturnal hypoxemia did not correlate with the hemodynamics for male patients, whereas SaO_2_ was a negative predictor for mPAP. T90% and mean SpO_2_ were independently associated with the hemodynamics for female patients. We developed an HSI that was combined with T90%, SaO_2_, and min SpO_2_ to distinguish CTEPH and CTEPD; HSI≥0.55 predicted the risk of CTEPH and exhibited 92.3% sensitivity and 87.5% specificity with an AUC of 0.937.

OSA has long been considered a predominantly male-related condition. A large cohort study (23) conducted at the end of the 20^th^ century in the United States showed that 24% males (*n* = 325) and 9% females (*n* = 250) had OSA when defined by an AHI>5. Other studies (14, 24) also proved an overall ratio of OSA ranging from 3:1 to 5:1 for males to females. However, a recent study (25) enrolled 400 females from a population-based random sample of 10,000 females aged 20–70 years and revealed 50% occurrence of OSA. The OSA prevalence of women increased with age (25, 26), and it may increase up to 75% between the age of 55 and 70 years. Our study showed that the prevalence of OSA in male and female patients were both higher (49 vs. 61%) than the general population, which may correlate with the disease condition (15). The mean age of female patients in our report was 63.40 years, and they had a light higher BMI than male patients, which likely explains why sex difference was not present in OSA for these patients. Furthermore, we also proved that OSA was not correlated with hemodynamics in both male or female patients, which was consistent with previous conclusions (27, 28). In addition, the third group of PH may have a higher prevalence of OSA than CTEPH or CTPED, in order to prevent the confound factor, patients with the coexisting of third and fourth group of PH had been excluded. But we can compare the SDB in CTEPH patients with or without chronic obstructive pulmonary disease in the future.

Nocturnal hypoxemia was proven highly prevalent in patients with CTEPH (18), but no sex difference was reported. In our cohort of CTEPH and CTEPD, we showed that female patients had worse nocturnal hypoxemia than male patients, and more female patients conformed to desaturation, though they had similar hemodynamics. In our study, we also noticed lower daytime oxygenation in female patients, which was significantly correlated with nocturnal hypoxemia. However, another study demonstrated that daytime oxygenation underestimates nocturnal oxygen desaturations in CTEPH implies other contributing factors for nocturnal hypoxemia. We further found distinctly different factors contributing to nocturnal hypoxemia between the sexes; for male patients, SaO_2_ was correlated with desaturation, whereas DLco-SB and PVR were associated with nocturnal hypoxemia and desaturation in female patients.

Female patients with acute PE present major pulmonary thromboembolic burden, more frequent right heart dysfunction, and may be more prone to developing CTEPH than men (2, 29). Mechanical compression of airway structure by small pulmonary arteries proliferating in adjacent lesions and residual thrombus may result in significant restrictive and obstructive ventilation dysfunction in patients with CTEPH (30–33). We found poorer pulmonary ventilation in female than male patients, which may be due to the fact that female patients also have higher thrombotic burden than male patients. Furthermore, pronounced functional impairment of the alveolocapillary membrane was found in CTEPH patients, and the reduction in pulmonary membrane diffusion capacity not only resulted in decreased diffusion capacity but also increased PVR (34). Although PVR in female patients was slightly higher than in male patients, the former group had significantly poorer DLco-SB in our study. Taken together, residual thrombotic burden appears to be a possible explanation for the sex difference of nocturnal hypoxemia in these patients.

Women with CTEPH had a more adaptive RV and therefore objective signs of disease was visible later in the disease course, resulting in relatively worse outcomes for women owing to the late diagnosis (3, 35). Another study (13) also showed sex-specific differences in cardiopulmonary function in CTEPH patients. The above points likely contribute to the sex difference of nocturnal hypoxemia in CTEPH.

Previous studies (18, 28, 36) concluded that mean SpO_2_ was associated with structural right ventricular remodeling and a poor prognosis in CTEPH patients. However, we found that T90% was correlated with mPAP in female patients, whereas mean SpO_2_ was independently correlated with PVR and CO. But in our study, we revealed that T90% was more important parameter in predicting hemodynamic than mean SpO_2_. T90% is the main component of HIS, and the sex difference of nocturnal hypoxemia and HSI may provide a hemodynamic prediction for CTEPH and CTEPD patients, but was only available for female patients. Future studies related to sex differences of nocturnal hypoxemia in CTEPH and CTEPD are warranted to confirm our findings and explore the prognosis of both sexes resulting from differences in nocturnal hypoxemia.

Our study has some limitations. First, it was designed as a single-center study, and the sample size of patients in each group was small, which may have caused a bias in our results. Our patients underwent the sleep study at varying time points following the diagnosis of CTEPH. Most CTEPH patients from NYHA-IV class had to be excluded because of severe daytime hypoxemia.

## Conclusion

Nocturnal hypoxemia was worse in female patients with CTEPH and CTEPD, whereas OSA was similarly prevalent between male and female patients. Nocturnal hypoxemia was correlated with the hemodynamics only in female patients. The HSI which a combination of T90%, SaO_2_, and min SpO_2_ showed high capacity for predicting CTEPH in females. Therefore, sleep-apnea monitoring needs to be more strongly promoted for female patients with CTEPH and CTEPD than male patients.

## Data availability statement

The original contributions presented in the study are included in the article/Supplementary material, further inquiries can be directed to the corresponding author/s.

## Ethics statement

The studies involving human participants were reviewed and approved by Local Institutional Ethics Committee of Shanghai Pulmonary Hospital (L22-198). The patients/participants provided their written informed consent to participate in this study.

## Author contributions

H-TLi, PY, Q-HZ, S-GG, and RJ conceived this research study and analyzed clinical data. J-LL, H-TLiu, H-LQ, W-HW, C-JL, and JH performed clinical management. H-TLi, PY, J-ML, and LW carried out manuscript organization, writing, and editing. All authors had full access to all the data in the study, take responsibility for the integrity of the data and the accuracy of the data analysis, and read and approved the final manuscript.

## Funding

This study was supported by grants from the Youth Program of National Natural Science Foundation of China (82000059), the Program of National Natural Science Foundation of China (81870042), and the National Natural Science Foundation of China Incubation Program (fkzr2147).

## Conflict of interest

The authors declare that the research was conducted in the absence of any commercial or financial relationships that could be construed as a potential conflict of interest.

## Publisher's note

All claims expressed in this article are solely those of the authors and do not necessarily represent those of their affiliated organizations, or those of the publisher, the editors and the reviewers. Any product that may be evaluated in this article, or claim that may be made by its manufacturer, is not guaranteed or endorsed by the publisher.
