# Supplementary material for: Sex-specific differences in sleep-disordered breathing and nocturnal hypoxemia in chronic thromboembolic pulmonary hypertension and chronic thromboembolic pulmonary disease
Source: Front Cardiovasc Med. 2022 Oct 17;9:966973. doi: 10.3389/fcvm.2022.966973 (PMC9618641; doi:10.3389/fcvm.2022.966973)
Supplement: Supplementary file 1 [file Table_1.DOCX]

Although chronic thromboembolic pulmonary hypertension (CTEPH) and chronic thromboembolic pulmonary disease (CTEPD) are known to be accompanied by symptoms associated with sleep-disordered breathing (SDB) and nocturnal hypoxemia, the sex-specific differences of SDB and nocturnal hypoxemia in patients with CTEPH and CTEPD remain unknown. We retrospectively collected 110 patients with CTEPH and CTEPD who underwent overnight cardiorespiratory polygraphy, firstly compared the characteristics of sleep-disordered breathing (SDB) and nocturnal hypoxemia between male and female patients with CTEPH and CTEPD patients, explored the sex-specific differences of SDB and nocturnal hypoxemia in patients with CTEPH and CTEPD, furtherly assessed the predictive value of sleep study indices to hemodynamic parameters and analyze the specific parameters to predict the risk of CTEPH; We have revealed that SDB was similarly present in males and females, and both sexes predominantly had obstructive sleep apnea (OSA); Sex-specific nocturnal hypoxemia was present in patients with CTEPH or CTEPD. 3. We also demonstrated that nocturnal hypoxemia was correlated with hemodynamics in female patients, furtherly developed a new composite parameter: hypoxemia scoring index (HSI), and the HSI showed high capacity for predicting the risk of CTEPH in females.
